# Supplementary material for: Direct translation of incoming retroviral genomes
Source: Nat Commun. 2024 Jan 5;15:299. doi: 10.1038/s41467-023-44501-7 (PMC10770327; doi:10.1038/s41467-023-44501-7)
Supplement: Supplementary file 1 — Supplementary Information [file 41467_2023_44501_MOESM1_ESM.pdf]

Supplementary information

Direct translation of incoming retroviral genomes

Julia Köppke, Luise-Elektra Keller, Michelle Stuck, Nicolas D. Arnow, Norbert Bannert, Joerg Doellinger, and Oya Cingöz*

* Corresponding author: cingoezo@rki.de

Supplementary Figure 1

Köppke *et al.*

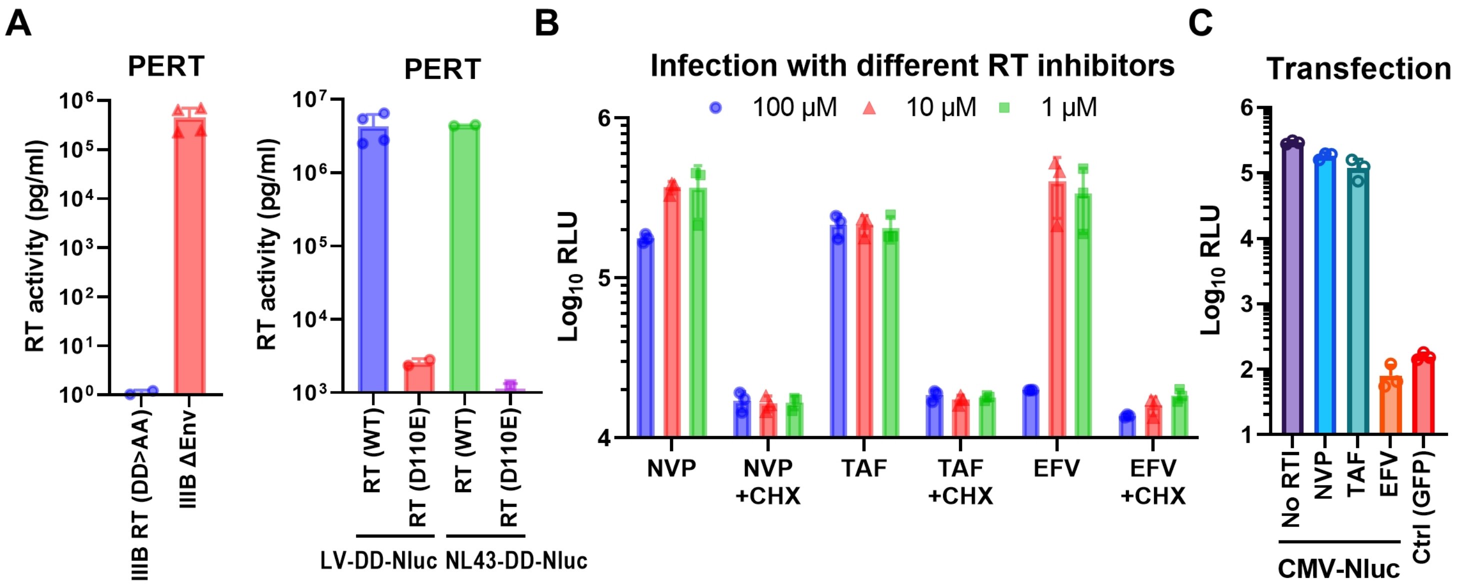

Supplementary Figure 1. Loss of RT activity by catalytic mutations or the use of different RT inhibitors.

(A) PERT assay on RT catalytic mutant virus HIV-1 IIIB (DD185/186AA) used in polysome fractionation, or the LV-DD-Nluc and NL43-DD-Nluc viruses produced with the RT catalytic mutant packaging vector (D110E) used in reporter assays. (B) Luciferase assay on cells transduced with LV-DD-Nluc virus in the presence of 1-100 µM of the RT inhibitors, with or without CHX. (C) Cells were transfected with a CMV-driven Nluc construct in the presence of 100 µM RT inhibitors. Toxicity was measured by luciferase assay 6 hours after transfection. NVP: Nevirapine, EFV: Efavirenz, TDF: Tenofovir disoproxil fumarate. Data represent mean +/- SD. Source data are provided as a Source Data file.

Supplementary Figure 2

Köppke *et al.*

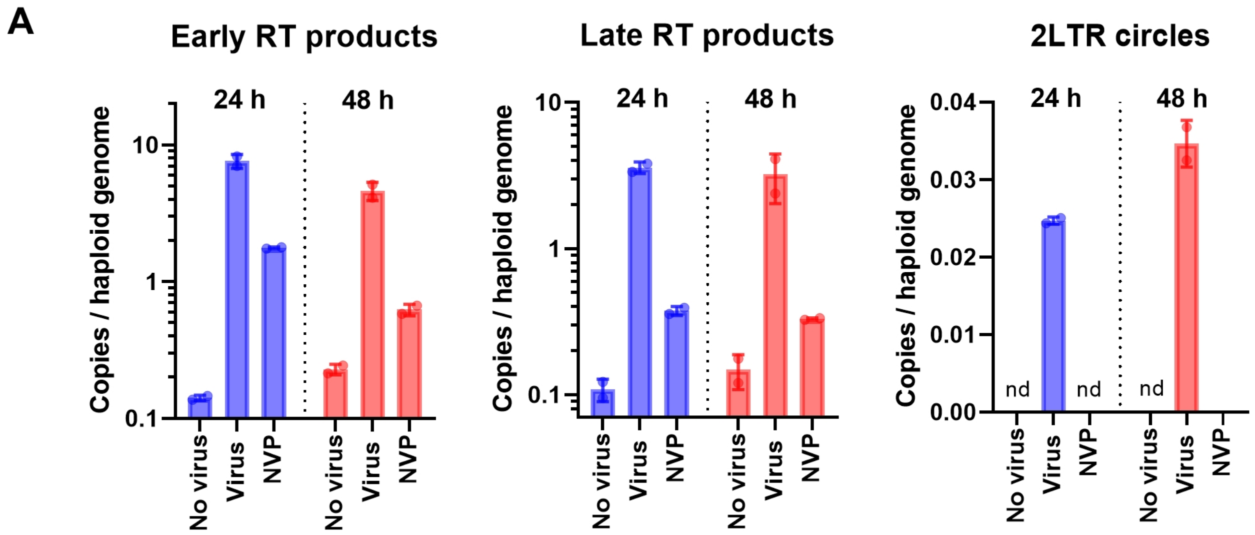

Supplementary Figure 2. Quantification of post-entry events after virus challenge. (A) 293T cells were infected with NL43-Firefly virus (40 ng/100K cells) and DNA was isolated at 24 and 48 hours post-infection. Early and late RT products, as well as 2-LTR circles were quantified by qPCR. Source data are provided as a Source Data file.

Supplementary Figure 3

Köppke *et al.*

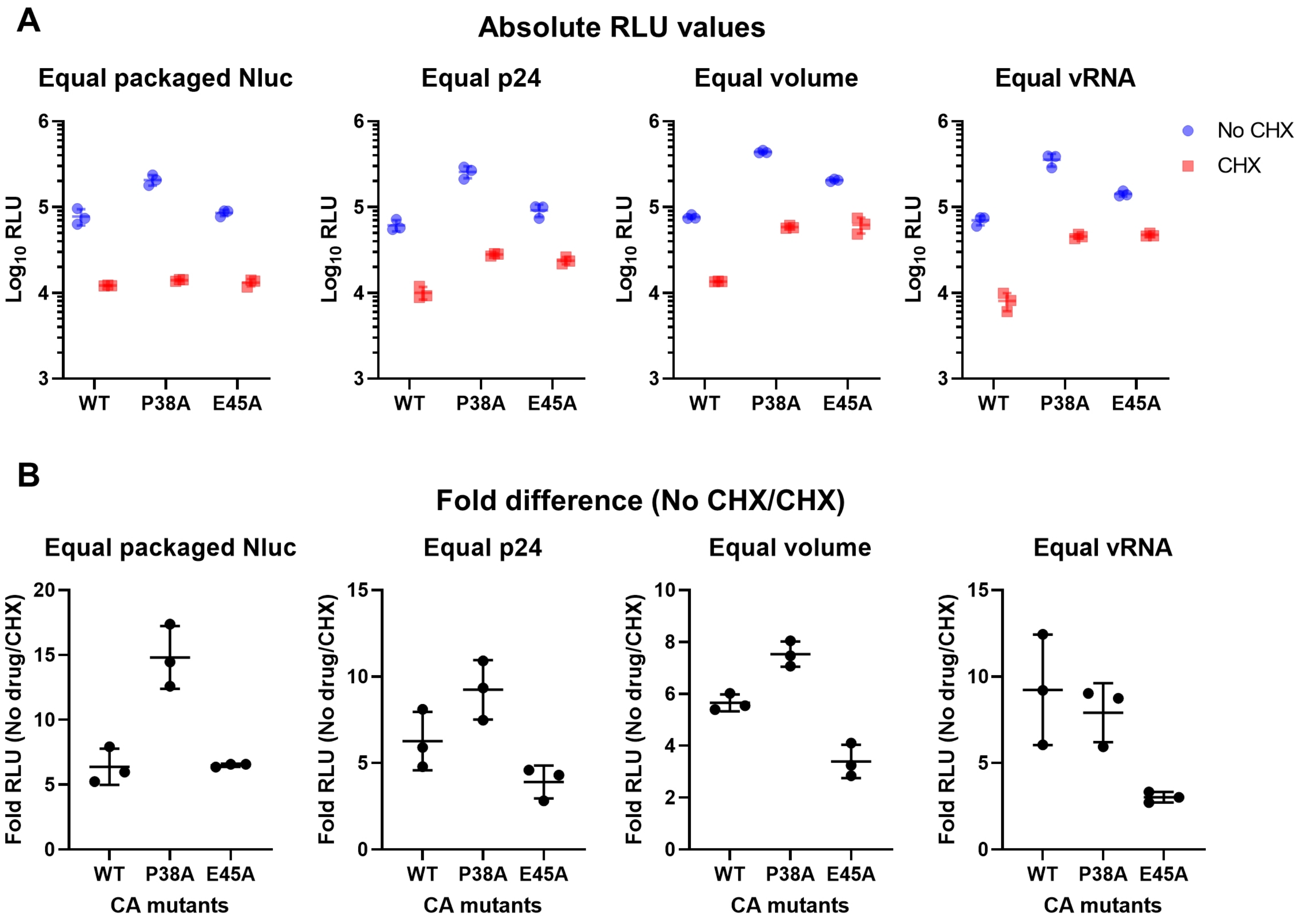

Supplementary Figure 3. Capsid stability mutations alter the translation potential of incoming retroviral genomes.

(A) Cells were infected with LV-DD-Nluc viruses containing WT, P38A or E45A capsids in the presence of NVP and Shield1, with or without CHX. Virus stocks were normalized based on either p24, viral RNA, virion-packaged Nluc or simple volume. (B) Fold difference between CHX-treated and untreated samples based on the data in (A). Data represent mean +/- SD. Source data are provided as a Source Data file.

Original Western Blots & Agarose Gels

Köppke *et al.*

Fig. 3B

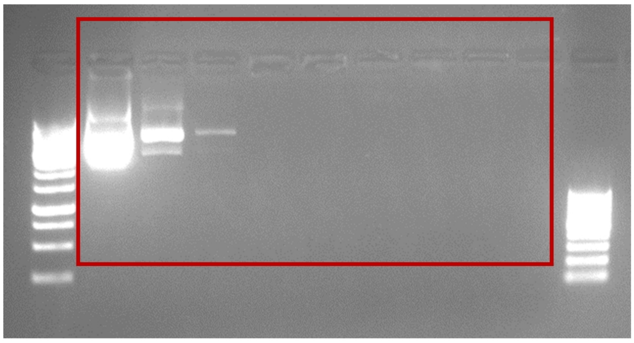

EtBr

Fig. 4C

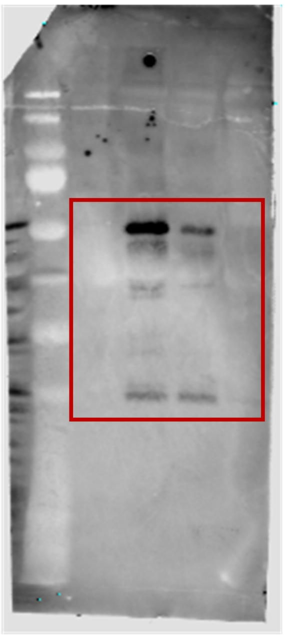

IP: α-p17+p24+55
IB: α-p24

Fig. 5D

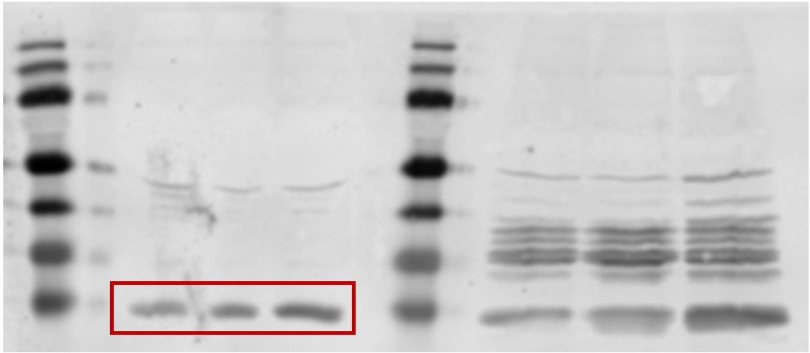

IB: α-p24
