# Supplementary material for: Direct translation of incoming retroviral genomes
Source: Nat Commun. 2024 Jan 5;15:299. doi: 10.1038/s41467-023-44501-7 (PMC10770327; doi:10.1038/s41467-023-44501-7)
Supplement: Supplementary file 3 — Reporting Summary [file 41467_2023_44501_MOESM3_ESM.pdf]

nature portfolio

Corresponding author(s): Oya Cingöz

Last updated by author(s): Dec 11, 2023

Reporting Summary

Nature Portfolio wishes to improve the reproducibility of the work that we publish. This form provides structure for consistency and transparency in reporting. For further information on Nature Portfolio policies, see our Editorial Policies and the Editorial Policy Checklist.

Statistics

For all statistical analyses, confirm that the following items are present in the figure legend, table legend, main text, or Methods section.

n/a	Confirmed	
☐	☒	The exact sample size (*n*) for each experimental group/condition, given as a discrete number and unit of measurement
☐	☒	A statement on whether measurements were taken from distinct samples or whether the same sample was measured repeatedly
☐	☒	The statistical test(s) used AND whether they are one- or two-sided<br>*Only common tests should be described solely by name; describe more complex techniques in the Methods section.*
☒	☐	A description of all covariates tested
☐	☒	A description of any assumptions or corrections, such as tests of normality and adjustment for multiple comparisons
☐	☒	A full description of the statistical parameters including central tendency (e.g. means) or other basic estimates (e.g. regression coefficient) AND variation (e.g. standard deviation) or associated estimates of uncertainty (e.g. confidence intervals)
☐	☒	For null hypothesis testing, the test statistic (e.g. *F*, *t*, *r*) with confidence intervals, effect sizes, degrees of freedom and *P* value noted<br>*Give P values as exact values whenever suitable.*
☒	☐	For Bayesian analysis, information on the choice of priors and Markov chain Monte Carlo settings
☒	☐	For hierarchical and complex designs, identification of the appropriate level for tests and full reporting of outcomes
☒	☐	Estimates of effect sizes (e.g. Cohen's *d*, Pearson's *r*), indicating how they were calculated

Our web collection on statistics for biologists contains articles on many of the points above.

Software and code

Policy information about availability of computer code

Data collection	No algorithms were used.
Data analysis	The LC-IMS-MS/MS data were analysed using FragPipe (version 20.0). Spectra were searched using MSFragger against the protein sequences of the human proteome (UP000005640, UniProtKB). Search results were validated using Percolator with MSBooster enabled rescoring and converged to false discovery rates of 1% on all levels.

For manuscripts utilizing custom algorithms or software that are central to the research but not yet described in published literature, software must be made available to editors and reviewers. We strongly encourage code deposition in a community repository (e.g. GitHub). See the Nature Portfolio guidelines for submitting code & software for further information.

Data

Policy information about availability of data

All manuscripts must include a data availability statement. This statement should provide the following information, where applicable:

- Accession codes, unique identifiers, or web links for publicly available datasets
- A description of any restrictions on data availability
- For clinical datasets or third party data, please ensure that the statement adheres to our policy

The mass spectrometry data generated in this study have been deposited in the public database PRIDE (https://www.ebi.ac.uk/pride/) under the accession number PXD046777. Source data are provided with this paper.

Research involving human participants, their data, or biological material

Policy information about studies with human participants or human data. See also policy information about sex, gender (identity/presentation), and sexual orientation and race, ethnicity and racism.

Reporting on sex and gender	The only human-derived samples used in this study was buffy coats form three anonymous healthy donors from the German Red Cross. Sex or gender was not considered in the study design.
Reporting on race, ethnicity, or other socially relevant groupings	This information has not been collected.
Population characteristics	This information has not been collected.
Recruitment	PBMCs were collected from the buffy coats of three anonymous healthy donors from the German Red Cross.
Ethics oversight	N/A

Note that full information on the approval of the study protocol must also be provided in the manuscript.

Field-specific reporting

Please select the one below that is the best fit for your research. If you are not sure, read the appropriate sections before making your selection.

[x] Life sciences [ ] Behavioural & social sciences [ ] Ecological, evolutionary & environmental sciences

For a reference copy of the document with all sections, see nature.com/documents/nr-reporting-summary-flat.pdf

Life sciences study design

All studies must disclose on these points even when the disclosure is negative.

Sample size	Sample size was chosen between n=2-12 (biological replicates) to generate data with sufficient statistical power.
Data exclusions	No data were excluded.
Replication	Typically, data represent replicates of three experiments. For polysome profiles and western blots, representative images are shown. Source data is provided with the paper.
Randomization	No randomization. Tests and controls were processed in parallel in the same experiment.
Blinding	No blinding.

Reporting for specific materials, systems and methods

We require information from authors about some types of materials, experimental systems and methods used in many studies. Here, indicate whether each material, system or method listed is relevant to your study. If you are not sure if a list item applies to your research, read the appropriate section before selecting a response.

Materials & experimental systems

n/a	Involved in the study	
[ ]	[x]	Antibodies
[ ]	[x]	Eukaryotic cell lines
[x]	[ ]	Palaeontology and archaeology
[x]	[ ]	Animals and other organisms
[x]	[ ]	Clinical data
[x]	[ ]	Dual use research of concern
[x]	[ ]	Plants

Methods

n/a	Involved in the study	
[x]	[ ]	ChIP-seq
[x]	[ ]	Flow cytometry
[x]	[ ]	MRI-based neuroimaging

Antibodies

Antibodies used	Anti-p24 antibody for ELISA and WB (AG3.0, NIH ARP #4121, mouse monoclonal and 183-H12-5C, NIH ARP-3537)<br>Anti-p55+24+17 antibody (Abcam, ab63917, rabbit polyclonal)
Validation	https://www.ncbi.nlm.nih.gov/pmc/articles/PMC6324184/

https://www.abcam.com/products/primary-antibodies/hiv1-p55--p24--p17-antibody-ab63917.html
https://www.licor.com/bio/reagents/irdye-800cw-goat-anti-mouse-igg-secondary-antibody
https://www.licor.com/bio/reagents/irdye-680rd-goat-anti-rabbit-igg-secondary-antibody

Eukaryotic cell lines

Policy information about cell lines and Sex and Gender in Research

Cell line source(s)	293T (F, CVCL_0063), Jurkat (M, CVCL_0065), and (CEM)A3.01 (F, CVCL_6244) were from ATCC. TZM-bl (F, CVCL_B478) and PM1 (M, CVCL_9472) were from the NIH HIV reagent program. THP-1 (M, CVCL_0006), U937 (M, CVCL_0007) and C8166 (M, CVCL_1099) were from internal laboratory stocks. PBMCs were isolated from buffy coats of three anonymous healthy donors from the German Red Cross.
Authentication	Some cell lines (293T, TZM-bl, THP-1, U937) were authenticated by STR testing from Eurofins Genomics. Others have not been tested.
Mycoplasma contamination	Some cell lines (Jurkat, PM1, A3.01) have tested negative for mycoplasma contamination by Eurofins Genomics. Others have not been tested.
Commonly misidentified lines (See ICLAC register)	None of the cell lines used in this study are listed in the ICLAC register v12.
